# Comparison of transvaginal cervical cerclage versus laparoscopic abdominal cervical cerclage in cervical insufficiency: a retrospective study from a single centre

**DOI:** 10.1186/s12884-022-05108-w

**Published:** 2022-10-17

**Authors:** Guiqiong Huang, Chunyan Deng, Hua Liao, Qing Hu, Haiyan Yu, Xiaodong Wang

**Affiliations:** 1 Department of Obstetrics and Gynecology, West China Second University Hospital, Sichuan University, No. 20, 3rd section, South Renmin Road, 610041 Sichuan, Chengdu, China; 2 Key Laboratory of Birth Defects and Related Diseases of Women and Children, Sichuan University, Ministry of Education, Chengdu, China

**Keywords:** Cervical insufficiency, Laparoscopic cervical cerclage, Transvaginal cervical cerclage, Pregnancy outcomes, Preterm birth, Twin pregnancy

## Abstract

**Background:**

Cervical cerclage has been proposed as an effective treatment for cervical insufficiency, but there has been controversy regarding the surgical options of cervical cerclage in singleton and twin pregnancies. This study aimed to compare the pregnancy outcomes between transvaginal cervical cerclage (TVC) and laparoscopic abdominal cervical cerclage (LAC) in patients with cervical insufficiency. We also aimed to evaluate the efficacy and safety, and provide more evidence to support the application of cervical cerclage in twin pregnancies.

**Methods:**

A retrospective study was carried out from January 2015 to December 2021. The primary outcomes were the incidence of spontaneous preterm birth (sPTB) < 24 weeks, < 28, < 32, < 34 weeks, and < 37weeks, gestational age at delivery, and the incidence of admission for threatened abortion or preterm birth after cervical cerclage. The secondary outcomes included admission to the Neonatal Intensive Care Unit, adverse neonatal outcomes and neonatal death. We also analysed the pregnancy outcomes of twin pregnancies after cervical cerclage.

**Results:**

A total of 289 patients were identified as eligible for inclusion. The LAC group (n = 56) had a very low incidence of sPTB ˂ 34 weeks, and it was associated with a significant decrease in sPTB < 28 weeks, ˂32 weeks, ˂34 and < 37 weeks, and admission to the hospital during pregnancy for threatened abortion or preterm birth after cervical cerclage (0 vs.27%; 1.8% vs. 40.3%; 7.1% vs. 46.8%; 14% vs. 63.5%, 8.9% vs. 62.2%, respectively; P < 0.001), and high in gestational age at delivery compared with the TVC group (n = 233) (38.3 weeks vs.34.4 weeks,P < 0.001). Neonatal outcomes in the LAC group were significantly better than those in the TVC group. The mean gestational age at delivery was 34.3 ± 1.8 weeks, with a total foetal survival rate of 100% without serious neonatal complications in twin pregnancies with LAC.

**Conclusion:**

In patients with cervical insufficiency, LAC appears to have better pregnancy outcomes than TVC. For some patients, LAC is a recommended option and may be selected as the first choice. Even in twin pregnancies, cervical cerclage can improve pregnancy outcomes with a longer latency period, especially in the LAC group.

**Supplementary Information:**

The online version contains supplementary material available at 10.1186/s12884-022-05108-w.

## Background

Cervical insufficiency (CI) is defined as the inability of the uterine cervix to retain a pregnancy without the signs and symptoms of clinical contractions and other clear pathology (e.g., bleeding, infection, ruptured membranes) in the second trimester or the early third trimester (< 34th weeks), which leads to late abortion or preterm birth [1]. The incidence rate of CI is estimated to be up to 1% of obstetric pregnancies [2], and CI accounts for recurrent spontaneous second trimester abortions and/or preterm births < 34 weeks [3]. Cervical cerclage (CC) has been proposed as an effective treatment for CI and has been used clinically for many years, thus successfully prolonging the pregnancy period and improving the perinatal prognosis [4]. The traditional surgical treatment is transvaginal cervical cerclage (TVC), which is straightforward and feasible. However, transabdominal cervical cerclage (TAC) may be performed when a TVC has failed in a previous pregnancy or in some cases with refractory cervical insufficiency [5]. TAC provides greater structural support to the cervix through placement of the suture at the internal os. Moreover, the absence of a foreign body in the vagina may reduce the risk of ascending infection and LAC can promote the ability to leave the suture in situ for future pregnancies, which is associated with a high rate of neonatal survival [6, 7]. With an increasing number of studies indicating that the prognosis of TAC is better than that of TVC, TAC has gradually become the primary abdominal approach [8]. However, there is no consensus regarding the first-line treatment for CI due to its advantages and limitations.

Especially in twin pregnancies, the American College of Obstetricians and Gynaecologists (ACOG) has claimed that in women with twin pregnancies and an ultrasonographically detected cervical length less than 25 mm, CC may increase the risk of preterm birth and is not recommended [1]. With the experience of CC in twin pregnancies, some retrospective cohort studies have found that perinatal outcomes are considerably improved in twin pregnancies [9], and emergency transvaginal cervical cerclage in twin women with cervical dilation and prolapsed membranes was associated with an overall 40% decrease in spontaneous preterm birth (sPTB) < 28 weeks of gestation and a prolongation of latency by 5 weeks [10]. A recent meta-analysis showed that for twin pregnancies with a cervical length less than 15 mm, CC was associated with a significant reduction in preterm birth [11]. Thus, what is the role of CC in twin pregnancies with CI in our hospital?

The objective of this study was to conduct an additional thorough investigation of the superiority and safety of LAC by comparing pregnancy and neonatal outcomes in patients with CI between the TVC and laparoscopic TAC (LAC) groups in a single centre. We also aimed to evaluate the efficacy and safety and to provide more evidence to support the application of CC in twin pregnancies with CI.

## Methods

### Study population

A retrospective study was conducted with medical records of pregnancies diagnosed with CI from January 2015 to December 2021 in the Department of Obstetrics, West China Second University Hospital in Sichuan, China, the regional tertiary referral centre. Approval was obtained from the Ethics Committee of West China Second Hospital, Sichuan University and informed consent was obtained from all participants. All cases of CI, either singleton or twin pregnancies with CC, were identified by reviewing the medical records of the hospital and telephone follow-up. Some patients had a typical history of ≥ 2 spontaneous second trimester abortions and/or preterm births < 34 weeks, and some patients with a typical history of ≥ 1 were found to have a spontaneous progressive reduction of the cervix on routine cervical length surveillance ultrasound. Other patients were diagnosed with cervical dilation and prolapsed membranes by transvaginal ultrasound or by pelvic examination because of mild symptoms such as vaginal discharge without obvious abdominal pain. Exclusion criteria included past pregnancy losses or preterm births due to an infection or induction, preterm premature rupture of membranes (PPROM) at diagnosis, obvious abdominal pain, active vaginal bleeding, clinical chorioamnionitis before the CC procedure, and foetal structural or genetic abnormalities. GA (gestational age) was determined by an evaluation of the last menstrual period and crown-rump length measurement on first-trimester ultrasound.

## Surgical techniques

Both TVC and LAC were carried out in our hospital, and no complications occurred during the procedures. The decision to perform cerclage and the treatment plan were made in consultation with patients. Before the procedures, vaginal and cervical swabs for microbiological analyses were obtained to rule out infection, and we excluded several contraindicated situations, such as active labour, placental abruption or PPROM.

TVC was performed by experienced obstetricians using the standardized transvaginal McDonald’s technique. Patients with emergency TVC at 17–25 weeks received broad-spectrum antibiotics, prophylactic intravenous tocolysis (ritodrine or atosiban) and magnesium sulphate (if necessary) before the procedure and for an additional 24 h. If they recovered well after surgery, patients were discharged to their home two days after CC and continued their prenatal care in the high-risk clinic. Prophylactic TVC was conducted at 12–18 weeks with no antibiotic or tocolytic prophylaxis. The onset of regular contractions, premature rupture of membranes, and/or suspicion of sepsis are indications for emergency removal of the cerclage. If the pregnancy course went smoothly, the cerclage was removed electively at 35–37 weeks gestation.

LAC was conducted electively by gynaecologists before pregnancy or during the first trimester. The surgical procedures of LAC were referred to previously published literature [12]. A nonabsorbable Mersilene tape (5 mm) with double needles was used for cervical cervix cerclage. In spite of the timing of placement, the surgical procedure performed was identical, except that a uterine probe (3 mm in diameter) was placed and fixed in the official cavity to delineate the cervicovaginal junction in non-pregnant women. With/without opening of the bladder peritoneum, each needle was inserted anteriorly to posteriorly using the uterosacral ligaments as landmarks at the level of the uterine isthmus, between the outer edge of the uterine isthmus and the medial to the uterine vessels. The uterine isthmus was ligated behind the uterine isthmus, and the uterine probe was removed at the end of the operation. Patients with LAC required a caesarean delivery to terminate the pregnancy. The transabdominal cerclage can be left in situ for future pregnancies or removed according to the will of the patients.

## Primary and secondary outcomes

The primary outcomes were the incidence of sPTB < 24 weeks, < 28, < 32, < 34 weeks, and < 37weeks, GA at delivery, and the incidence of admission for threatened abortion or preterm birth after CC (admission after CC). The main secondary outcomes included new-born birth weight, delivery-related complications (such as cervical laceration, postpartum haemorrhage, and/or clinical chorioamnionitis), 1-min Apgar score < 7, 5-min Apgar score < 7, admission to the NICU, adverse neonatal outcomes (respiratory distress syndrome, necrotizing enterocolitis, intraventricular haemorrhage, sepsis, retinopathy of prematurity requiring laser therapy and bronchopulmonary dysplasia) and neonatal death. We also analysed the pregnancy outcomes of twin pregnancies with CI after CC.

### Statistical analysis

Data are described by the mean ± standard deviation, number [%] or median (interquartile range). Continuous variables were compared using the Student’s t test (for normally distributed data) or the Mann–Whitney U test (for nonnormal distribution). Categorical-type outcomes were analysed with the chi-square test or Fisher’s exact test. A P value < 0.05 was considered statistically significant. Data analysis was performed by SPSS version 24.0 for Windows (SPSS Inc., Chicago, IL, USA).

## Results

### Patient characteristics

A total of 289 patients were identified as eligible for inclusion during the study period, among which 233 women accepted TVC and 56 underwent LAC. There was no difference in the majority characteristics of the two groups (Table 1). The mean age of the TVC group and LAC group was 30.96 ± 4.12 years vs. 32.27 ± 3.89 years, *P* = 0.28. Most of the patients (79.9%) had a typical history of ≥ 1 spontaneous second trimester abortions and/or preterm births < 34 weeks. Some patients received CC because of ultrasonographic cervical progressive reduction during pregnancy after prior operative hysteroscopy, prior cervical surgery, and/or routine examination in twin pregnancies. The LAC group had more prior failed transvaginal cerclage procedures and more spontaneous second trimester abortions and/or preterm births < 34 weeks than the TVC group (10.7% vs. 1.7%, *P* = 0.005; 1 (0.5-2) vs. 1 (1–2), *P* < 0.001). A total of 160 patients in the TVC group underwent emergency cervical cerclage, compared with none in the LAC group. In the TVC group, the median length of the cervical canal was 1.2 cm in the emergency group and 2.12 cm in the prophylactic CC group. In the LAC group, 47 patients underwent prepregnant LAC and nine in the first trimester (8–10 weeks). The median gestational age at cerclage placement was 22.4 weeks in the TVC group. No perioperative complications (e.g., bleeding > 100 mL, infection, injury to bowel or bladder) occurred in either group. Six patients in the TVC group received repeat cerclage due to membrane bulging or funnelling below the knots (details shown in Table 2), while all patients in the LAC group received only one transabdominal cerclage.

**Table 1:** Clinical characteristics of the study population

	TVC group	LAC group	*P*
Patients (n)	233	56	-
Age(years)	30.96 ± 4.12	32.27 ± 3.89	0.28
Gravidity	3(2–4)	3(2–5)	0.044
the incidence of twin pregnancies (n[%])	46 [19.7%]	7 [12.5%]	0.209
Prior ssA-PTB	1(0.5-2)	1(1–2)	< 0.001
Prior failed transvaginal cerclage	4[1.7%]	6[10.7%]	0.005
Prior operative hysteroscopy (n[%])	53[23%]	8[14%]	0.164
Prior cervical surgery (n[%])	11[5%]	5[10%]	0.216
Conception by IVF(n[%])	68[29%]	19[34%]	0.487
Emergency cervical cerclage (n[%])	160[68%]	0[0]	< 0.001
GA at cerclage placement (weeks)	22.4 (17-24.7)	before pregnancy or during the first trimester	-
Bleeding during the CC (ml)	15.6 ± 6.8	20.3 ± 5.2	0.37
The length of the cervix (cm)	1.2 (0.8-2)	-	-
Emergency CC	1 (0.6–1.5)		
prophylactic CC	2.12 (1.5-3.0)	-	
Cervical dilation at diagnosis (cm)	0(0-0.5)	-	-

TVC: transvaginal cervical cerclage; LAC: laparoscopic abdominal cervical cerclage; ssA-PTB: spontaneous second-trimester abortions and/or preterm births < 34 weeks; IVF: In-Vitro Fertilization; CC: cervical cerclage

**Table 2:** The detailed data of 6 patients received repeat cerclage in the TVC group

	GA at first CC	GA at repeat CC	Gravidity	prior ssA-PTB	Prior hysteroscopy	GA at delivery	Delivery Mode	complications	Apgar score (1-5-10 min)
patient 1	25 + 2	28 + 2	6	2	0	28 + 5	VD	/	8-9-9
patient 2	16 + 4	25 + 3	3	2	1	37 + 4	VD	CL, PPH	10-10-10
patient 3	14 + 5	22 + 4	4	2	0	29 + 3	CD	/	8-9-9
patient 4	20 + 3	23 + 5	6	1	1	26 + 6	VD	CL	7-8-9
patient 5	18	24 + 1	3	1	6	28	VD	CL, PPH	8-9-9
patient 6	20 + 5	25 + 1	3	1	1	28 + 4	CD	CL	8-9-10/7-8-9

TVC: transvaginal cervical cerclage; GA: gestational age; CC: cervical cerclage; ssA-PTB: spontaneous second-trimester abortions and/or preterm births; VD: vaginal delivery; CD: caesarean delivery; CL: cervical laceration; PPH: postpartum haemorrhage

## Pregnancy outcomes

For the primary outcome, 15 (6.4%) patients with sPTB < 24 weeks of gestation occurred due to the failure of TVC, while there was one patient who unfortunately had intrauterine foetal death at 21 weeks in the LAC group. The incidence of sPTB < 28, < 32, < 34 and < 37 weeks was significantly less common in the LAC group than in the TVC group (Table 3). The median GA at delivery was 38.3 weeks in the LAC group, which was higher than that in the TVC group, and there was statistical significance between the two groups. In the TVC group, 148 (63.5%) patients were admitted to the hospital for threatened abortion or preterm birth after CC, and only eight patients were in the other group (*P* < 0.001).

**Table 3:** The primary outcomes between the TVC group and the LAC group

	TVC group (n = 233)	LAC group (n = 56)	P
sPTB < 24 weeks	15[6.4%]	0	0.08
sPTB < 28 weeks	63[27%]	0	< 0.001
sPTB < 32 weeks	94[40.3%]	1[1.8%]	< 0.001
sPTB < 34 weeks	109[46.8%]	4[7.1%]	< 0.001
sPTB < 37 weeks	145 [62.2%]	15 [8.9%]	< 0.001
GA at delivery	34.4(27.1–38.7)	38.3(36-39.6)	< 0.001
Admission after CC(n[%])	148[63.5%]	8[14%]	< 0.001

TVC: transvaginal cervical cerclage; LAC: laparoscopic abdominal cervical cerclage; sPTB: spontaneous preterm birth; GA: estational age; CC: cervical cerclage

In the LAC group, one patient had intrauterine foetal death at 21 weeks and then underwent laparoscopic surgery to remove the tape for vaginal delivery. For the remaining patients, a caesarean delivery was performed to terminate the pregnancy in the third trimester with no complications, and 58.2% (32/55) decided to keep the cerclage in situ for future pregnancies. In the TVC group, most of the patients underwent vaginal delivery, and 93 patients underwent caesarean delivery, with a surgery rate of 39.9% (93/233). However, 26 patients had delivery-related complications, such as cervical laceration, postpartum haemorrhage, and/or clinical chorioamnionitis in the TVC group, which was significantly greater than the occurrence in the LAC group (15.9% vs. 0, *P* = 0.009. Table 4).

**Table 4:** The secondary outcomes between the TVC group and the LAC group

	TVC group (n = 233)	LAC group (n = 56)	P
Cesarean delivery(n[%])	93[39.9%]	55[98.2%]	< 0.001
Duration of hospital stay after delivery (days)	3(2–4)	3(3–4)	< 0.001
removal of the cerclage (n[%])	233 [100%]	24 [42.8%]	< 0.001
delivery-related complications^®^ (n[%])	37 [15.9%]	0	< 0.001
Infection and Clinical chorioamnionitis	12 [5.2%]	0	0.132
Neonate births	274 + 5*	60 + 3*	-
Stillbirths (n[%])	29 [10.6%]	1 [1.7%]	0.029
Live births (n[%])	245 [89.4%]	59 [98.3%]	0.029
Birth weight (kg)	2.21 ± 0.99	3.01 ± 0.63	0.000
NICU admission (n[%])	141 [57.6%]	10 [16.9%]	0.000
1-min Apgar score	10 (8–10)	10 (10–10)	< 0.001
5-min Apgar score	10 (9–10)	10 (10–10)	< 0.001
1-min Apgar score < 7	35 [14.3%]	0	0.002
5-min Apgar score < 7	9 [3.7%]	0	0.214
Duration of neonatology stay (days)	29 (9–55)	9 (5.75-19)	0.017
Neonate death	14 [5.7%]	0	0.08

®the include ; *means selective reduction of multifetal pregnancies

TVC: transvaginal cervical cerclage; LAC: laparoscopic abdominal cervical cerclage; NICU: Neonatal Intensive Care Unit

Apart from 8 patients with selective reduction of multifoetal pregnancies, one patient in the LAC group encountered intrauterine foetal death in the second trimester, and 29 patients suffered from stillbirth in the TVC group due to TVC failure. The total foetal survival rate in the two groups was 91.0% (304/334), of which the rate was higher in the LAC group than in the TVC group, with no significance (98.3% vs. 89.4%, *P* = 0.183. Table 4). The mean new-born birth weight and the Apgar score (1 and 5 min) were higher, and the NICU admission rate was lower in the LAC group than in the TVC group, with significant differences. Similarly, the LAC group had better neonatal outcomes and fewer neonatal complications than the TVC group (Table 4, Supplemental Table 1). However, there was no difference in the 5-min Apgar score < 7, necrotizing enterocolitis, sepsis or neonatal death between the two groups. To reduce the interference of cervical length, we performed a subgroup analysis between the prophylactic CC and LAC groups, and found similar pregnancy outcomes (Table 5, Supplemental Table 2).

**Table 5:** A subgroup analysis between prophylactic CC and LAC group

	prophylactic TVC group (n = 73)	LAC group (n = 56)	P
sPTB < 24 weeks	4	0	0.132
sPTB < 28 weeks	11	0	0.002
sPTB < 32 weeks	17	1	< 0.001
sPTB < 34 weeks	22	4	0.001
GA at delivery	36.4 (32.6–37.7)	38.3(36-39.6)	< 0.001
Admission after CC(n[%])	48 [65.7%]	8[14%]	< 0.001
Caesarean delivery(n[%])	28	55	< 0.001
Length of hospital stay after delivery (days)	2 (2–3)	3(3–4)	< 0.001
delivery-related complications (n[%])	12 [16.4%]	0[0]	0.001
Infection and Clinical chorioamnionitis	3 [4.1%]	0	0.257
Neonate births	76	60 + 3*	-
Stillbirths (n[%])	12 [15.8%]	1 [1.7%]	0.006
Live births (n[%])	64 [84.2%]	59 [98.3%]	0.001
Birth weight (kg)	2.43 ± 1.00	3.01 ± 0.63	< 0.001
NICU admission (n[%])	28 [43.8%]	10 [16.9%]	0.001
1-min Apgar score	10 (8–10)	10 (10–10)	< 0.001
5-min Apgar score	10 (8–10)	10 (10–10)	< 0.001
1-min Apgar score < 7	8 [12.5%]	0	0.006
5-min Apgar score < 7	3 [4.7%]	0	0.245
Duration of neonatology stay (days)	37 (7–52)	9 (5.75-19)	0.04
Neonate death	3 [4.7%]	0	0.245

*means selective reduction of multifetal pregnancies

CC: cervical cerclage; LAC: laparoscopic abdominal cervical cerclage; sPTB: spontaneous preterm birth; GA: gestational age; VD: vaginal delivery; NICU: Neonatal Intensive Care Unit

## Twin pregnancies

In the case of cervical cerclage in twin pregnancies in the TVC group, most (95.7%, 44/46) of the patients received operations at a cervical length of < 1.5 cm. Forty-one of 46 patients in the TVC group had an emergency CC due to a progressive reduction of the cervix, and some had ≥ 1 prior spontaneous second trimester abortions and/or preterm births < 34 weeks. A total of 97.8% of patients had a prior operative hysteroscopy history. GA at delivery ranged from 23.6 to 38 weeks: three patients at < 24 weeks, 31 between 24 and 33.9 weeks, and 12 ≥ 34 weeks (4 ≥ 37 weeks). The mean GA at delivery was 30.7 ± 4.5 weeks gestation, with a median gestational latency of 7.8 weeks. Apart from six neonate deaths due to extremely preterm birth and three due to selective reduction, we obtained 83 live new-borns with a mean birth weight of 1.58 kg.

In the LAC group, our study included seven women with two monochorionic/diamniotic and five dichorionic/diamniotic twin pregnancies. They underwent prophylactic TAC due to CI before pregnancy (n = 4) or during the first trimester (n = 3). The obstetrical and neonatal outcomes are presented in Table 6. GA at delivery ranged from 32 to 37 weeks (6 patients at < 37 weeks), and the mean GA at delivery was 34.3 ± 1.8 weeks. There were 12 live new-borns with a mean birth weight of 2.1 kg. The NICU admission rate in the LAC group was 58.3%, mostly due to GA < 34 weeks and/or birth weight < 2.0 kg, but no serious neonatal complications were reported in all new-borns.

**Table 6:** The pregnancy outcomes of twin pregnancies

	TVC group	LAC group
patients (n)	46	7
Prior sPTB	0 (0–1)	1 (1–2)
Prior operative hysteroscopy (n[%])	45 [97.8%]	0
Conception by IVF(n[%])	36 [78.3%]	5 [71.4%]
Rescue cervical cerclage (n[%])	41 [89.1%]	-
The length of the cervix < 1.5 cm	44 [95.7%]	-
The length of the cervix (cm)	0.8 (0.5–1.5)	-
Cervical dilation at diagnosis (cm)	0 (0–1)	-
Gestational latency (weeks)	7.8 (4.9–11.5)	-
GA at delivery	30.7 ± 4.5	34.3 ± 1.8
sPTB < 24 weeks	3	0
sPTB < 28 weeks	13	0
sPTB < 32 weeks	25	0
sPTB < 34 weeks	34	3
sPTB < 37 weeks	42	6
Live births (n[%])	83 [90.2%]	12 [100%] + 2*
Birth weight (kg)	1.58 ± 0.65	2.1 ± 0.44
NICU admission (n[%])	69 [83.1%]	7 [58.3%]

*means selective reduction of multifetal pregnancies

TVC: transvaginal cervical cerclage; LAC: laparoscopic abdominal cervical cerclage; sPTB: spontaneous preterm birth; IVF: In-Vitro Fertilization; GA: gestational age; NICU: Neonatal Intensive Care Unit

## Discussion

Cervical insufficiency (CI) is one of the main reasons for late abortion or preterm birth, which increases the burden on families and society as a whole. In women with CI, the recurrence rate of second trimester delivery was 72% in women with no cerclage, 30% in women with prophylactic TVC, and 5% in women with TAC [13], which means CC has been shown to be effective in the treatment of CI. Previous research found that CC prevents preterm birth in singleton women with a previous preterm birth once a short cervix has been detected on ultrasound imaging [14].

In the present study, we analysed 289 patients with CI, in which 233 women underwent TVC during pregnancy and 56 received LAC. The choice of procedure was dependent on the clinician on duty/performing the procedure and the patients after they were informed about both procedures. Most of the patients who had a typical history of ≥ 2 prior spontaneous second trimester abortions and/or preterm births < 34 weeks would like to seek medical advice before pregnancy or during the first trimester and then receive LAC or prophylactic TVC. The traditional concept is the preference for TVC for CI patients as first-line treatment while considering the suitability of TAC, either open TAC or LAC, for patients who were diagnosed with refractory cervical insufficiency or who had a prior failed TVC suture [5, 15]. It is known that the main disadvantage of TAC is the caesarean delivery to terminate the pregnancy and the complications during the TAC procedure. Nevertheless, with the development of laparoscopic techniques, some new approaches for LAC may be considered as an acceptable alternative to traditional LAC due to its superiority in terms of tranvaginal removal [16]. Surgeons have had extensive experience in ensuring that the procedure is successful and minimally invasive [17]. In our study, there were no adverse events, and all patients were discharged within 24-48 h in the TAC group. Moreover, TAC avoids the infection risk of vaginal surgery and movement restrictions after surgery, and patients can take care of their duration of pregnancy by themselves. A recent systematic review extrapolated that LAC is a reasonable alternative to open TAC and may be preferable because of benefits such as cosmesis and recovery [18]. Therefore, an increasing number of researchers prefer to concentrate on the benefits of LAC before pregnancy or during the first trimester.

The currently available literature provides much evidence that indicates the superiority of pregnancy outcomes and neonatal outcomes in the LAC group to TVC. As a regional tertiary referral centre, we perform many cases with cervical cerclage, including TVC and LAC. However, there has been little analysis of the data in our hospital in recent years. Several findings in our study are notable. (1) Because previous studies have shown that TAC is suitable for patients who had a prior failed TVC suture, and may be associated with a lower risk of perinatal death or delivery at < 24 weeks [15, 19], such patients are prone to choose TAC. Therefore, the LAC group had more prior failed transvaginal cerclage procedures than the TVC group. (2) The LAC group had a very low incidence of sPTB < 34 weeks, and it was associated with a significant decrease in sPTB < 28 weeks, < 32, < 34 and < 37 weeks, and admission to the hospital during pregnancy for threatened abortion or preterm birth after CC and high in GA at delivery in comparison to the TVC group. (3) Neonatal outcomes in the LAC group were significantly better than those in the TVC group. These results in our retrospective study suggest that LAC is more effective than TVC. We speculate that the reasons for this result include the following. (1) The cerclage was positioned in the cervico-isthmic area, in a more proximal position in the LAC group, resulting in its stability. There were six patients in the TVC group who received a repeat cerclage (three prophylactic CCs at the first cerclage), while all patients in the LAC group received only one transabdominal cerclage, which is consistent with the retrospective study demonstrating that LAC is associated with better preservation of the cervical length throughout pregnancy than TVC [20]. (2) The timing of the LAC before pregnancy period avoids adverse effects of surgical stimulation on pregnancy. (3) Cervical shortness is one of the causes of sPTB. The patients in the TVC group had a shorter cervical length at cerclage placement than those in the LAC group, which may be associated with poorer pregnancy outcomes. However, it is worth noting that we obtained the same outcomes when we performed a subgroup analysis between the prophylactic CC and LAC groups without the interference of cervical length. (4) Transvaginal surgery has a higher risk of infection than transabdominal surgery, and infection is an essential indicator for termination of pregnancy. Although there was no significant difference between groups, there were 12 (5.2%) patients with infection and clinical chorioamnionitis in the TVC group, which is much greater than that in the LAC group where none occurred.

A number of retrospective studies have reported pregnancy and neonatal outcomes for LAC before pregnancy or during the first trimester [21, 22]. Whittle et al. reported data for 65 patients with LAC and found that the foetal salvage rate (n = 67 pregnancies) was 89% with a mean gestational age of 35.8 ± 2.9 weeks [23]. Chen et al. showed a series of 101 LAC cases with an average GA at delivery of 36.2 weeks, a 95% foetal survival rate and no complications [24]. Ades et al. demonstrated that all patients with LAC in their study delivered via caesarean delivery with an average gestational age of 37.1 weeks [25]. In our report, the median GA at delivery in the LAC group was 38.3 weeks, with a 93.6% foetal survival rate and low NICU admission. Moreover, the finding that only a small portion of patients were admitted to the hospital for threatened abortion or preterm birth after CC during pregnancy in the LAC group can reduce the financial and emotional burden on patients and families and solve the problem of increased risk of venous thromboembolism due to movement restrictions. Meanwhile, prior research reported that when left in situ for subsequent pregnancies, laparoscopic transabdominal cerclage is associated with a high rate of neonatal survival [7]. Therefore, for some patients, could LAC be selected as the first choice?

There are some different opinions on whether TVC can be recommended in multiple pregnancies for preventing preterm birth. Some studies have shown no current evidence of a benefit for TVC was found in multiple pregnancies, and an increased risk of PTB, very low birth weight and respiratory distress [1, 26−27]. However, in another retrospective study, it was concluded that in twin pregnancies with a cervix ≤ 15 mm before 24 weeks, CC was associated with a significant prolongation of pregnancy by almost 4 more weeks and significantly decreased preterm birth < 34 weeks and admission to the NICU compared with controls [28]. In a recent study, Wu et al. found that ultrasound-indicated TVC in dichorionic/diamniotic twin pregnancies could decrease PTB, especially for women with a cervical length < 15 mm [29]. Zeng et al. reported that emergency cerclage with the standardized transvaginal McDonald’s technique in twin women with cervical dilation and prolapsed membranes was associated with better pregnancy outcomes [10]. We briefly analysed the pregnancy outcome of CC in twin pregnancies. In our study, 44 patients had cervical lengths < 15 mm with a median 7.8-week latency period from CC to delivery in the TVC group. Our results, including GA at delivery and neonatal outcomes, compare favourably to previously reported series that reported that the mean (min.- max.) gestational age at delivery was 27.3 (21–34) weeks, and the median time between cervical cerclage and delivery was 6.4 weeks [30]. The results indicating the potential to consider offering CC to twin pregnancies with cervical shortening (< 15 mm) corroborate those of previous reports.

A case series and literature review showed 80% of pregnancies with transabdominal cerclage delivered beyond 32 weeks and 35% after 37 weeks gestation with an overall perinatal survival of 91% and without adverse events [9]. in the LAC group, all of our cases delivered ≥ 32 weeks with a total foetal survival rate of 100%, providing good obstetric results without increasing perioperative morbidity and good evidence for the view that TAC efficiently suppresses the risk of sPTB for patients with CI in twin pregnancies.

One of the limitations in our study is the unbalanced patients number between the two groups (233 vs. 56). We need larger prospective controlled studies about laparoscopic abdominal cervical cerclage to further confirm its superiority, especially in twin pregnancies.

## Conclusion

To conclude, we found that LAC, as a more effective treatment for CI patients, appears to have better pregnancy outcomes than TVC. Moreover, our limited experience suggested that LAC before pregnancy or during the first trimester is a recommended option for CI patients. Even in twin pregnancies, CC can improve pregnancy outcomes with a longer latency period, especially in the LAC group. Given the high efficacy and low harmfulness, LAC may be used as a first-line treatment in certain indications, such as in patients who have a typical history of ≥ 2 prior spontaneous second trimester abortions and/or preterm births < 34 weeks or that are willing to select LAC even if they have a typical history of ≥ 1 after they understand the advantages and limitations. Of course, larger prospective controlled studies are needed for confirmation.

## Electronic supplementary material

Below is the link to the electronic supplementary material.

**Supplementary Material 1**

**Supplementary Material 2**

## Data Availability

The datasets used and/or analyzed during the current study are available from the corresponding author on reasonable request.

## List of abbreviations

TVC: Transvaginal cervical cerclage.
TAC: Transabdominal cervical cerclage.
LAC: Laparoscopic abdominal cervical cerclage.
CI: Cervical insufficiency.
CC: Cervical cerclage.
sPTB: Spontaneous preterm birth.
GA: Gestational age.
ACOG: American College of Obstetricians and Gynecologists.
PPROM: Preterm premature rupture of membranes.
NICU: Neonatal Intensive Care Unit.
