# Supplementary material for: Comparison of transvaginal cervical cerclage versus laparoscopic abdominal cervical cerclage in cervical insufficiency: a retrospective study from a single centre
Source: BMC Pregnancy Childbirth. 2022 Oct 17;22:773. doi: 10.1186/s12884-022-05108-w (PMC9575299; doi:10.1186/s12884-022-05108-w)
Supplement: Supplementary file 1 — Supplementary Material 1 [file 12884_2022_5108_MOESM1_ESM.doc]

**supplemental data 1** The adverse neonatal outcomes between the TVC group and the LAC group

|  | TVC group (n=233) | LAC group (n=56) | P |
| --- | --- | --- | --- |
| Respiratory distress syndrome | 72 [29.4%] | 6 [10%] | 0.002 |
| Necrotizing enterocolitis | 11 [4.5%] | 1[1.7%] | 0.473 |
| Intraventricular hemorrhage | 33 [13.5%] | 2 [3.4%] | 0.029 |
| Sepsis | 25 [10.2%] | 3 [5.1%] | 0.222 |
| Retinopathy of prematurity | 41 [16.7%] | 0 | <0.001 |
| Bronchopulmonary dysplasia | 35 [14.3%] | 0 | <0.001 |

TVC: transvaginal cervical cerclage; LAC: laparoscopic abdominal cervical cerclage.
