# Supplementary material for: Comparison of transvaginal cervical cerclage versus laparoscopic abdominal cervical cerclage in cervical insufficiency: a retrospective study from a single centre
Source: BMC Pregnancy Childbirth. 2022 Oct 17;22:773. doi: 10.1186/s12884-022-05108-w (PMC9575299; doi:10.1186/s12884-022-05108-w)
Supplement: Supplementary file 2 — Supplementary Material 2 [file 12884_2022_5108_MOESM2_ESM.doc]

**Supplemental Table 2** The adverse neonatal outcomes between prophylactic CC and LAC group

|  | prophylactic TVC group (n=73) | LAC group (n=56) | P |
| --- | --- | --- | --- |
| Respiratory distress syndrome | 14 [21.9%] | 6 [10%] | 0.091 |
| Necrotizing enterocolitis | 1 [1.6%] | 1[1.7%] | 1 |
| Intraventricular hemorrhage | 4 [5.3%] | 2 [3.4%] | 0..681 |
| Sepsis | 8 [12.5%] | 3 [5.1%] | 0.15 |
| Retinopathy of prematurity | 6 [7.9%] | 0 | 0.028 |
| Bronchopulmonary dysplasia | 7 [9.5%] | 0 | 0.014 |

CC: cervical cerclage; LAC: laparoscopic abdominal cervical cerclage.
